# A Self‐Assembled MOF‐*Escherichia Coli* Hybrid System for Light‐Driven Fuels and Valuable Chemicals Synthesis

**DOI:** 10.1002/advs.202308597

**Published:** 2024-04-25

**Authors:** Jialu Li, Junfeng Shen, Tianfeng Hou, Hongting Tang, Cuiping Zeng, Kemeng Xiao, Yanping Hou, Bo Wang

**Affiliations:** ^1^ CAS Key Laboratory of Quantitative Engineering Biology Shenzhen Institute of Synthetic Biology Shenzhen Institute of Advanced Technology Chinese Academy of Sciences Shenzhen 518055 China; ^2^ School of Resources Environment and Materials Guangxi University Nanning 530004 China; ^3^ Department of Chemistry and Center for Cell and Developmental Biology The Chinese University of Hong Kong Shatin Hong Kong 999077 China

**Keywords:** electrostatical assembly, *Escherichia coli*, lysine synthesis, metal–organic frameworks, semi‐artificial photosynthesis

## Abstract

The development of semi‐artificial photosynthetic systems, which integrate metal–organic frameworks (MOFs) with industrial microbial cell factories for light‐driven synthesis of fuels and valuable chemicals, represents a highly promising avenue for both research advancements and practical applications. In this study, an MOF (PCN‐222) utilizing racemic‐(4‐carboxyphenyl) porphyrin and zirconium chloride (ZrCl_4_) as primary constituents is synthesized. Employing a self‐assembly process, a hybrid system is constructed, integrating engineered *Escherichia coli* (*E. coli*) to investigate light‐driven hydrogen and lysine production. These results demonstrate that the light‐irradiated biohybrid system efficiently produce H_2_ with a quantum efficiency of 0.75% under full spectrum illumination, the elevated intracellular reducing power NADPH is also observed. By optimizing the conditions, the biohybrid system achieves a maximum lysine production of 18.25 mg L^−1^, surpassing that of pure bacteria by 332%. Further investigations into interfacial electron transfer mechanisms reveals that PCN‐222 efficiently captures light and facilitates the transfer of photo‐generated electrons into *E. coli* cells. It is proposed that the interfacial energy transfer process is mediated by riboflavin, with facilitation by secreted small organic acids acting as hole scavengers for PCN‐222. This study establishes a crucial foundation for future research into the light‐driven biomanufacturing using *E. coli*‐based hybrid systems.

## Graphical abstract

A self‐assembly biohybrid is constructed by combining *Escherichia coli* with a metal‐organic framework via multiple interactions. Under illumination, the system demonstrates a remarkable improvement in H_2_ production and NADPH level, leading to an increased production of NADPH‐dependent lysine. Mechanistic investigations indicate riboflavin plays a role in electron transfer, while secreted small organic acids act as hole scavengers and carbon source.

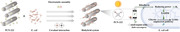

## 1 Introduction

Solar‐driven fuel and chemical production is of paramount importance for advancing the sustainable development of human society.^[^ ^1^, ^2^ ^]^ Photosynthesis, nature's primary pathway for solar‐to‐chemical energy conversion, underpins vital resources such as food, medicine, fuel, and materials.^[^ ^3^ ^]^ Despite its fundamental role, photosynthetic organisms frequently encounter inefficiencies stemming from suboptimal light‐harvesting and conversion processes.^[^ ^4^ ^]^ In response, recent decades have witnessed the exploration of semiconductor materials that possess the dual capability of both light harvesting and catalytic reactions.^[^ ^5^ ^]^ These artificial photosynthetic systems have demonstrated remarkable solar‐to‐chemical energy conversion efficiencies, surpassing those achieved by natural photosynthetic organisms.^[^ ^3^, ^6^, ^7^ ^]^ However, the utilization of semiconductor materials in catalytic processes is hindered by their inherent lack of the high selectivity demonstrated by biological enzymes, making the synthesis of complex multi‐carbon valuable products a rare achievement.^[^ ^7^ ^]^ To address this, semi‐artificial photosynthetic systems (SAPS) have emerged, employing semiconductor materials for light harvesting and biological enzymes or cells as catalytic centers. This innovative approach capitalizes on the synergistic strengths of both components,offering a powerful strategy to enhance overall performance.^[^ ^8^ ^]^ In a pivotal study, Yang et al. used light‐induced reduction of CO_2_ to acetic acid by employing cadmium sulfide (CdS) nanoparticles deposited on the surface of acetogenic bacteria.^[^ ^9^ ^]^ Following this breakthrough, CdS has found application in numerous other microorganisms, driving further exploration in SAPS.^[^ ^10^, ^11^, ^12^ ^]^ However, CdS is prone to photo‐corrosion, and the ensuing release of Cd^2+^ ions poses significant biological and environmental risks.^[^ ^13^ ^]^ Recent studies in SAPS have also investigated alternative semiconductor materials like g‐C_3_N_4_, InP, TiO_2_, and quantum dots, each showing varying levels of improvement in stability and biosafety.^[^ ^14^, ^15^, ^16^, ^17^, ^18^ ^]^ Nevertheless, there remains room for enhancement, particularly in terms of achieving precise size control and optimizing light‐harvesting capabilities of photosensitive materials.^[^ ^8^ ^]^

Metal–organic frameworks (MOFs) represent a burgeoning class of functional materials, formed through the synergistic combination of metal ions and organic ligands.^[^ ^19^ ^]^ Distinguished by their crystalline structure and well‐defined pores, MOFs boast significantly larger specific surface areas compared to conventional porous materials.^[^ ^20^ ^]^ The incorporation of organic components imparts them with versatile design capabilities, enabling for the customization of pore sizes and facile functionalization of pore surfaces.^[^ ^19^ ^]^ An MOF shell composed of Zr_6_O_4_(OH)_4_(BTB)_2_(OH)_6_(H_2_O)_6_, exhibiting catalytic activity for decomposing reactive oxygen species (ROS), was utilized to encapsulate a strictly anaerobic acetogenic bacterium. The implementation of this innovative approach led to a fivefold reduction in the bacterial death rate when exposed to air with an oxygen concentration of 21%, enabling the bacteria to continuously produce acetic acid through CO_2_ fixation even under oxidative stress conditions.^[^ ^21^ ^]^ A recent study in SAPS revealed that light‐irradiated MOF UiO‐66 could enhance the intracellular reducing power of *Clostridium butyricum*, resulting in a notable increase in butyric acid production.^[^ ^22^ ^]^ Interestingly, compared to other semiconductor materials, the use of MOFs as light‐harvesting agents for bacteria remains a relatively underexplored area.^[^ ^23^ ^]^

In recent years, industrial microorganisms, particularly *Escherichia coli* and *Saccharomyces cerevisiae* have emerged as favored choices among researchers for the construction of biohybrid systems dedicated to light‐driven fuel and chemical production.^[^ ^16^, ^17^, ^24^, ^25^, ^26^ ^]^ This preference arises from their remarkable versatility in synthesizing diverse products and their adaptability to genetic engineering techniques.^[^ ^27^, ^28^ ^]^ For instance, we have successfully established several light‐driven H_2_ evolution hybrid systems based on *E. coli*.^[^ ^10^, ^29^, ^30^, ^31^, ^32^, ^33^ ^]^ Beyond hydrogen, these systems have also demonstrated the production of various valuable organic compounds including acids, alcohols, and biofuels.^[^ ^30^, ^31^, ^34^ ^]^ Despite these achievements, the precise mechanism underlying interfacial electron transfer between semiconductor materials and *E. coli* cells remains a subject of ongoing investigation. This knowledge gap represents a significant barrier to the optimization and systematic engineering of future biohybrid systems.^[^ ^36^, ^37^ ^]^

Lysine, an indispensable amino acid crucial for human health, is an endogenous product synthesized by *E. coli*.^[^ ^35^ ^]^ Its significance extends beyond nutrition, finding critical applications in both medical and chemical domains.^[^ ^37^ ^]^ The biomanufacturing process of lysine necessitates a substantial intracellular energy supply in the form of NADPH.^[^ ^38^ ^]^ Consequently, the development of a light‐driven lysine synthesis strategy in *E. coli* holds substantial promise in research and application potentials. Previous studies have revealed that MOF materials containing porphyrin or metalloporphyrin exhibit exceptional light capture efficiency and catalytic activity.^[^ ^39^, ^40^ ^]^ Furthermore, MOF materials based on zirconium oxide (ZrO_2_) clusters have gained significant prominence in the field of photocatalysis research.^[^ ^41^, ^42^ ^]^ In this study, we synthesized an MOF (PCN‐222) and explored the combination of PCN‐222 and *E. coli* to form a self‐assembled hybrid system. Initially, we assessed the performance of light‐driven H_2_ evolution to validate the efficacy of the biohybrid system. The light‐driven lysine production was systematically investigated based on an engineered *E. coli* with enhanced NADPH supply and lysine accumulation. Finally, we delved into the energy transfer processes to elucidate potential mechanisms at the material–cell interface. This study conclusively demonstrates that PCN‐222 can effectively act as a photoelectron donor for non‐photosynthetic industrial microbial cell factories, enabling light‐driven synthesis of fuels and valuable chemicals.

## 2 Results and Discussion

**Figure 1:**
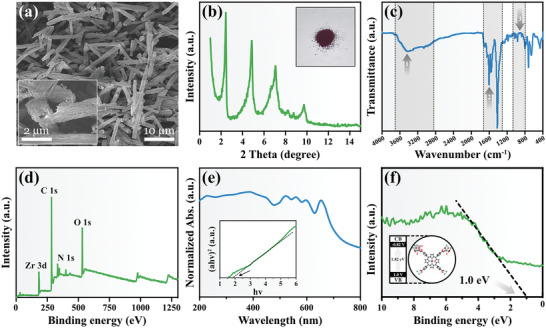
Characterization of PCN‐222. a) SEM images of PCN‐222 (Mag = 2k×). b) XRD patterns and photographic image (inset) of PCN‐222. c) FTIR spectra of PCN‐2 × 2. d) XPS survey spectra of PCN‐222. e) UV–vis–DRS absorption spectra and tauc plot (inset) of PCN‐222. f) VB and energy band structure (inset) of PCN‐222.

PCN‐222 was synthesized via a hydrothermal method, as outlined in the Experimental section.^[^ ^42^ ^]^ A comprehensive investigation was undertaken to analyze the morphology and physicochemical properties of PCN‐222. In **Figure** **1**a, scanning electron microscope (SEM) images of PCN‐222 at various magnifications reveal a distinctive hollow rod‐like structure, with lengths spanning from 10 to 20 µm. Notably, the material's surface exhibits a relatively rough texture, creating a favorable environment for bacterial adhesion.^[^ ^43^ ^]^ The PCN‐222 powder exhibits a distinctive purple‐black hue, and X‐ray diffraction (XRD) analysis of PCN‐222 revealed characteristic diffraction peaks at 2.44°, 4.86°, 7.08°, 8.24°, and 9.72°, respectively (Figure 1b), consistent with the simulated model of the PCN‐222 crystal structure.^[^ ^42^ ^]^ Moreover, the Fourier transform infrared spectroscopy (FTIR) spectrum exhibited a broad peak at 3500 cm^−1^ in the FTIR spectrum (Figure 1c), corresponding to the stretching vibration peak of ─OH/─NH_2_ (indicated by arrow 1). Furthermore, a distinct vibration peak in the range of 1400 to 1800 cm^−1^ was observed, corresponding to the C═N─C vibration peak in PCN‐222 (arrow 2), consistent with previous literature reports.^[^ ^44^ ^]^ The vibration peak observed between 800 and 1000 cm^−1^ corresponds to the ─COOH functional group (arrow 3), confirming the presence of the (4‐carboxyphenyl) porphyrin (TCPP) compound within the prepared PCN‐222 structure. The chemical composition of PCN‐222 was thoroughly investigated using X‐ray photoelectron spectroscopy (XPS) (Figure 1d). The XPS spectra spanning from 0 to 1300.0 eV revealed characteristic binding energy features corresponding to Zr 3d, C 1s, N 1s, and O 1s. Figure S1a (Supporting Information) displays the high‐resolution Zr 3d spectrum of PCN‐222. The main peak at 182.26 eV is attributed to the Zr─O bond. The carboxyl groups exhibit a notable affinity for Zr (IV), promoting the formation of stable MOF structures. The higher binding energy indicates the presence of extremely strong zirconium–oxygen coordination bonds. The dominant peak at 184.61 eV is attributed to the Zr 3d_3/2_‐Zr^4+^ element orbital, confirming the Zr valence state in the MOF structure as +4.^[^ ^45^ ^]^ The C 1s spectrum (Figure S1b, Supporting Information) displays a peak at 284.38 eV, characteristic of C─C bonds, and another peak at 288.19 eV, corresponding to C═N─C bonds, which aligns with the observations in the N 1s spectrum (Figure S1c, Supporting Information). In the XPS spectrum of O 1s (Figure S1d, Supporting Information), the peak at 531.22 eV corresponds to the Zr─O bond, while the peak at 529.45 eV is attributed to the C─O bond. These findings are consistent with the analyses reported in previous studies.^[^ ^46^ ^]^ Together, these results confirm the successful synthesis of PCN‐222, utilizing TCPP and zirconium chloride as primary constituents. The Ultraviolet–visible diffuse reflectance spectroscopy (UV–vis–DRS) analysis of PCN‐222 revealed its absorption capability within the 200–700 nm range (Figure 1e), signifying its potential for efficient charge separation upon excitation by visible light. The bandgap energy of PCN‐222 was determined through the tauc plot transformed from UV–vis–DRS (Figure 1e) and estimated to be 1.82 eV, in agreement with prior research.^[^ ^47^ ^]^ The position of the valence band (VB) was determined through X‐ray valence band spectroscopy (Figure 1f) and was measured to be 1.0 eV. Accordingly, the conduction band position was calculated to be −0.82 eV. This energy band structure indicates that PCN‐222 can effectively capture sunlight (<681 nm) and generate high‐energy electrons (Figure 1f). Given that the potential for numerous intracellular redox reactions falls below −0.7 eV, the photogenerated electrons from PCN‐222 have the potential to initiate a wide array of biochemical reactions in *E. coli*.^[^ ^48^ ^]^

**Figure 2:**
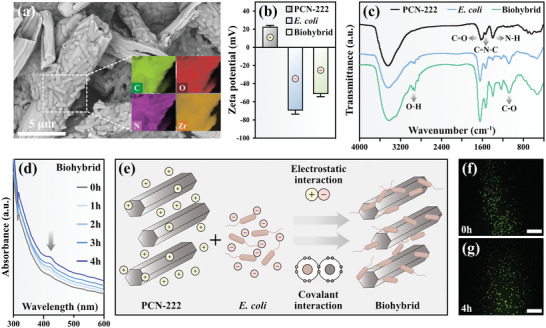
Characterization of the biohybrid. a) SEM images of *E. coli* cells combined with PCN‐222 (Mag = 25k×) and elemental mapping of C, N, O, and Zr (inset). b) Zeta potentials of PCN‐222, *E. coli*, and biohybrid. c) FTIR spectra of PCN‐222, *E. coli*, and biohybrid in water. d) Absorbance spectra of biohybrid within 4 h. e) Light Schematic illustrations of the assembly process of biohybrid. f) Staining fluorescence image of biohybrid before 24 h dark reaction, scale bar 10 µm. g) Staining fluorescence image of biohybrid after 24 h dark reaction, scale bar 10 µm. Fluorescent dyes: propidium iodide (PI) and fluorescein Isothiocyanate (FITC).

The surface of PCN‐222 exhibited a remarkable accumulation of *E. coli* cells, as evidenced by SEM analysis (**Figure** **2**a). The surface morphology of PCN‐222‐*E. coli* hybrid system (biohybrid) was also distinctly discerned using atomic force microscopy (AFM). Comparatively, the biohybrid displayed a surface where numerous *E. coli* cells enveloped the PCN‐222 material, a feature not observed in groups with pure bacteria or pure material (Figure S2, Supporting Information). These observations highlight the interaction between the *E. coli* cells and PCN‐222 in the biohybrid system. To delve further into the binding interaction between the materials and bacteria, the surface potential was subsequently assessed. The surface potentials of PCN‐222 and *E. coli* were measured at 22.7 and −69.58 mV, respectively (Figure 2b), signifying the potential for electrostatic interaction between the MOF material and bacterial cells. After subjecting PCN‐222 and *E. coli* to a 24‐h incubation in agitated culture medium (detailed in the Experimental Section), the surface potential of the resulting mixture was recorded at −51.36 mV. This observation suggests the formation of the biohybrid was through electrostatic assembly (Figure 2b).

In addition to electrostatic interactions, covalent interactions may also be at play due to the abundant functional groups on the surface of the MOF surface.^[^ ^49^, ^50^ ^]^ Consequently, the functional groups on the surfaces of the materials, bacteria, and biohybrid were analyzed using infrared spectroscopy (Figure 2c). It was observed that the positions of C═O, C═N─H, and N─H on the biohybrid exhibited slight shifts compared to pure PCN‐222, indicating potential interactions of these functional groups with *E. coli* cells. Furthermore, the presence of the C─O peak in both the biohybrid and *E. coli* cells, while absent in PCN‐222, further supports the effective integration of PCN‐222 with *E. coli*. Owing to the presence of chromophores, characterized by unsaturated bonds such as C═O bonds, on the surface of PCN‐222, and auxochromes, which comprise saturated groups containing heteroatoms like O─H bonds on the surface of *E. coli*, their combination leads to a hyperchromic effect.^[^ ^51^ ^]^ This effect enhances the molar extraction coefficient, thereby leading to an increase in absorbance.^[^ ^52^ ^]^ The absorption spectrum of pure bacteria exhibits a smooth curve, while PCN‐222 displays a small peak around 420 nm owing to the presence of unsaturated bonds (Figure S3, Supporting Information). As time progresses, the peak strength of the biohybrid at 420 nm gradually intensifies, providing evidence of an increasingly prominent hyperchromic effect (Figure 2d). This observation further substantiates the notion that the binding interaction between *E. coli* and PCN‐222 strengthens progressively with time. Based on the experimental findings outlined above, Figure 2e illustrates the self‐assembly process of *E. coli* and PCN‐222. The surface of PCN‐222 bears a positive charge, whereas the surface of *E. coli* is negatively charged. This electrostatic charge difference leads to their spontaneous assembly through electrostatic adsorption. Additionally, covalent interactions are identified, further intensifying and stabilizing the integration of the biohybrid.

Given the pivotal role of material biocompatibility in bacterial activity, we conducted a thorough investigation into the toxicity of PCN‐222 on *E. coli* cells. Figure 2f and Figure S4a (Supporting Information) illustrate the favorable activity of both *E. coli* and the biohybrid system prior to the reaction. Subsequently, Figure 2g and Figure S4b (Supporting Information) reveal a slight decrease in bacterial activity following the dark reaction. However, it is important to note that the proportion of viable bacteria remains high within the overall bacterial population, with a survival rate exceeding 95% experiences a notable decline after 24 h of light exposure. Notably, in the coating experiment, the biohybrid group exhibits a minimal decrease in bacterial count compared to the pure bacteria group following 24 h of dark reaction. Even when the PCN‐222 concentration in the system was increased to 10 mg, the bacteria maintained satisfactory activity after 24 h of reaction. These compelling findings provide strong evidence for the outstanding biocompatibility of PCN‐222. In the light experiment, as depicted in Figure S5 (Supporting Information), it is evident that the colony survival rate remains unaffected in the system with the addition of 1, 3, and 5 mg when compared to the control group. However, in the systems with the addition of 7.5 and 10 mg, the colony survival rate decreases to below 50%. This observation suggests that the increase in material content may lead to excessive production of ROS within the system under light conditions, subsequently resulting in bacterial mortality.^[^ ^21^ ^]^

**Figure 3:**
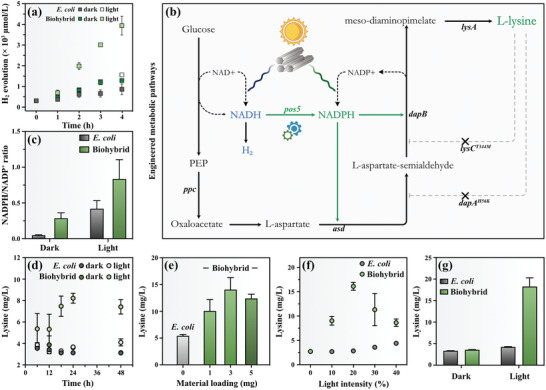
Investigation on lysine production by biohybrid and pure *E. coli* under light and dark. a) H_2_ evolution of pure wild‐type *E. coli* and biohybrid in the dark versus light (38.93 mW cm^−2^). b) illustration of engineered *E. coli* lysine synthesis pathway engineering. c) NADPH/NADP^+^ ratios of engineered *E. coli* and biohybrid in the dark versus light (38.93 mW cm^−2^). d) Lysine production of engineered *E. coli* at different time points (55.7 mW cm^−2^). e) Lysine production of *E. coli* BL21(DE3) with different concentrations of materials loading (55.7 mW cm^−2^). f) Lysine production of engineered *E. coli* under different light intensity (20% light intensity = 38.93 mW cm^−2^). g) Lysine production of engineered *E. coli* under optimal conditions (38.93 mW cm^−2^); (wild‐type *E. coli*: *E. coli* MG1655, engineered *E. coli*: *E. coli* BL21 (DE3); (*ppc*: encoding phosphoenolpyruvate carboxylase (PEPCx); *asd*: encoding aspartate‐semialdehyde dehydrogenase (ASADH); *lysC^T344M^*: encoding aspartokinase (AK) III; *lysA*: encoding diaminopimelate decarboxylase (DAPDC); *dapA^H56K^*: encoding dihydrodipicolinate synthetase (DHDPS); *dapB*: encoding dihydrodipicolinate reductase (DHDPR); *pos5*: NADH kinase).

Our previous studies on *E. coli*‐based hybrid systems have elucidated that surplus reducing power, generated by semiconductor materials through photoelectrons, is discharged as H_2_ to uphold cellular redox equilibrium.^[^ ^30^ ^]^ Therefore, *E. coli*‐based hybrid systems could be an ideal solar‐to‐fuel conversion platform. To verify the effectiveness of the system, we first used wild‐type *E. coli* MG 1655 to demonstrate the effectiveness of the system. Then we initiated our study by quantifying the biohydrogen yield from the resulting MOF‐*E. coli* hybrid system under dark and light conditions. **Figure** **3**a illustrates that, after 4 h, the light‐irradiated biohybrid group exhibited a markedly elevated H_2_ (3865.7 µmol L^−1^) compared to the other groups. This compelling result underscores the occurrence of effective interfacial energy transfer in the presence of both the material and light. Furthermore, it's noteworthy that the H_2_ evolution from pure PCN‐222 was found to be negligible. The apparent quantum efficiency for H_2_ evolution at 38.93 mW cm^−2^ was measured at 0.75% (the calculation process is shown in the Experimental Section). In comparison to other semi‐artificial photosynthetic systems (as depicted in Table S1, Supporting Information), the quantum yield of our system is relatively modest. This observation could be attributed to the specific structure and properties of the photosensitive materials employed, as well as the light intensity utilized within the semi‐artificial photosynthetic system.^[^ ^53^ ^]^ Furthermore, the negative surface potential of microorganisms may pose challenges for efficient transmission of photogenerated electrons from the materials to the microorganisms. Nevertheless, it is noteworthy that the achieved quantum efficiency of 0.75% exceeds that of certain well‐established algae and plants, indicating the promising potential of our system.^[^ ^7^ ^]^ These results illustrated that the photoelectrons generated by PCN‐222 under light could be effectively transferred into *E. coli* cells. Previous study has found that overexpression of *ppc*, *lysC^T344M^*, *asd*, *dapA^H56K^*, *dapB*, and *lysA* (the enzymes encoded by these genes are marked in the legend in Figure 3) can promote lysine synthesis in *E. coli*.^[^ ^53^ ^]^ Based on this study, we engineered the lysine synthesis pathway of *E. coli* BL21(DE3) and the conversion of NADH to NADPH by *pos5* fragment from *S. cerevisiae* in *E. coli* was enhanced, then we utilized the engineered *E. coli* to construct the biohybrid for subsequence experiments (Figure 3b). Figure S8 (Supporting Information) presents the hydrogen production curve of the engineered *E. coli*, highlighting notable differences between the light and dark groups. The light group exhibits a substantial increase in hydrogen production compared to the dark group, while the material group demonstrates a slight improvement compared to the pure bacteria group. However, when compared to the hybrid system incorporating wild‐type *E. coli*, the engineered *E. coli* shows a significant reduction in hydrogen production capacity. This outcome can be attributed to the introduction of the *pos5* gene from *Saccharomyces cerevisiae* through gene engineering. The encoded enzyme, NADH kinase, plays a crucial role in the de novo synthesis of NADPH, thereby redirecting the energy generated through metabolism toward product synthesis rather than hydrogen production.^[^ ^7^ ^]^ Consequently, the hydrogen production capacity of the engineered *E. coli* experiences a substantial decrease or even becomes negligible, as depicted in Figure S9 (Supporting Information).

The biosynthesis of lysine in engineered *E. coli* is intricately tied to the availability of intracellular NADPH.^[^ ^56^, ^57^ ^]^ Therefore, the NADPH/NADP^+^ ratio in *E. coli* was assessed in the presence and absence of PCN‐222. Figure 3c demonstrates that the NADPH/NADP^+^ ratio of the biohybrid was 2.0 times and 2.93 times higher than that of the pure bacteria under light and in the dark, respectively. These findings indicate that the photoelectrons generated by PCN‐222 significantly accelerated the circulation of NADPH, which is beneficial for the synthesis of lysine. It is worth noting that the NADPH/NADP^+^ ratio of pure *E. coli* under light conditions was higher compared to that in the dark. This difference can be attributed to the intracellular response of bacteria to light irradiation.^[^ ^58^ ^]^ Consequently, this led to an augmentation in lysine production by engineered *E. coli* in the light control group in contrast to the dark control group. Subsequently, we delved into the light‐driven lysine synthesis performance of the biohybrid, considering various reaction times, material concentrations, and light intensities. Notably, the lysine production exhibited a rapid increase after 12 h, culminating at a peak of 8.22 mg L^−1^ after 24 h (Figure 3d). Further sampling at 24 h revealed that 3 mg of PCN‐222 had the maximum enhancement effect on lysine production under light, reaching a value of 14.04 mg L^−1^, which is 2.6 times that of the pure bacteria group (5.4 mg L^−1^) (Figure 3e). Building upon the established optimal material concentration, we proceeded to investigate the impact of light intensity, spanning from 0% to 40%, on the system. Notably, the lysine production in the biohybrid exhibited an ascending trend, reaching its zenith at 20% light intensity (20% light intensity = 38.93 mW cm^−2^) with an impressive yield of 16.11 mg L^−1^. This value stands at 5.69 times that of the pure bacteria group, which achieved a lysine content of 2.83 mg L^−1^ (Figure 3f). In the pure *E. coli* group, a slight increase in lysine production was observed with the rise in light intensity. This phenomenon can be attributed to the photosensitizing effect of reducing equivalents, as previously confirmed in *E. coli*‐C_3_N_4_ hybrid systems.^[^ ^18^ ^]^ However, in contrast to the pure bacterial group, the biohybrid group exhibited a decline in lysine production performance with further increments in light intensity. This observation is consistent with previous literature, which reports that high light intensity may induce cell stress and trigger the generation of harmful reactive oxygen species.^[^ ^59^ ^]^ Despite the observed decline in performance at high light intensities, the lysine productivity of the biohybrid remained significantly superior to that of the pure *E. coli*. This suggests that the energy derived from PCN‐222 could still be efficiently transferred to *E. coli* cells even under stress. Guided by these insights, we systematically optimized the conditions for light‐driven lysine production in the biohybrid, conducting multiple repetitions of the experiments (Figure 3g). Remarkably, the biohybrid achieved a lysine productivity of 18.25 mg L^−1^, signifying a remarkable 332% increase compared to pure bacteria (4.22 mg L^−1^). These compelling results firmly establish the capacity of PCN‐222 to substantially enhance lysine synthesis under light conditions. To further verify the feasibility of this system, we tested its lysine production every 24 h within 3 days and found that although lysine production showed a decreasing trend with increasing days, the production on the third day remained above 70% of the first day (Figure S10, Supporting Information). After conducting it testing on the hybrid material before and after 24 h of illumination, it was found that its photoelectric performance did not show significant attenuation (Figure S11, Supporting Information), indicating that this system has a certain degree of stability.

**Figure 4:**
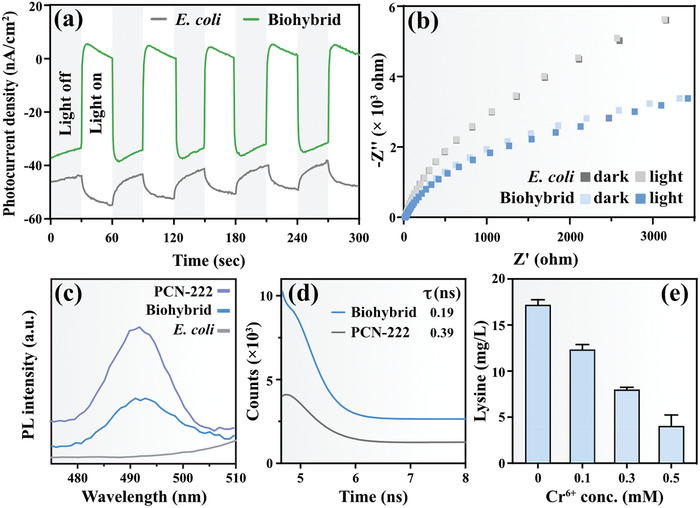
Study on interface electron transfer of the biohybrid. a) Photocurrent response of biohybrid and *E. coli*. b) Nyquist plots of biohybrid and *E. coli* under dark and light conditions. c) The steady‐state PL spectra of PCN‐222, biohybrid and pure *E. coli* cells. d) TRPL of biohybrid and PCN‐222. e) Lysine production of biohybrid with the addition of electron scavengers.

With the established optimal conditions for light‐driven lysine production in place, we proceeded to the subsequent of our study, focusing on unraveling the interfacial electron transfer mechanism within the biohybrid. Initially, we undertook tests to evaluate the photoelectrochemical properties of both pure *E. coli* and the biohybrid. As shown in **Figure** **4**a, the fast, stable and reproducible photocurrent response of biohybrid in repeated dark/light cycle experiments implied that the biohybrid has good photo‐responsiveness. In contrast, *E. coli* exhibited the opposite response to the biohybrid. This discrepancy can be attributed to the competitive adsorption and interception of the secreted redox organic compounds by *E. coli*.^[^ ^60^ ^]^ The semi‐arc of the electrochemical impedance Nyquist spectrum for the biohybrid was found to be smaller than that of *E. coli* (as shown in Figure 4b), both under dark and light conditions. These findings that PCN‐222 increases the electronic conductivity of the system, which is conducive to rapid interfacial charge transfer. Upon exposure to light, a noticeable reduction in the radius of the biohybrid was observed, indicating the occurrence of charge transfer at the material–cell interface. Subsequently, the electrochemical impedance spectra of both pure bacteria and biohybrid were meticulously analyzed. Figure S12 (Supporting Information) illustrates the corresponding equivalent circuit employed for the analysis, while Table S2 (Supporting Information) provides the precise values of the relevant resistance parameters. Remarkably, a notable reduction in impedance is observed in the biohybrid when compared to pure *E. coli*. This substantial decrease serves as clear evidence that the electron transfer rate within the biohybrid system is significantly accelerated. These findings underscore the capacity of the combined PCN‐222 and *E. coli* system to effectively diminish charge transfer resistance, consequently facilitating the efficient transfer of photogenerated charges at the interface. Collectively, these findings provide robust evidence supporting the conclusion that the biohybrid system is conducive to efficient charge‐carrier separation and migration. To confirm that the PCN‐222 can effectively transfer the captured light energy to *E. coli*, electron transfer at the material–cell interface was investigated using photoluminescence (PL). As depicted in Figure 4c, no noticeable fluorescence signal was detected within the *E. coli* cells. In contrast, the PL intensity of the biohybrid was notably lower than that of PCN‐222. This observation strongly indicates that PCN‐222 operates as an electron donor while *E. coli* serves as an electron acceptor, effectively mitigating the electron–hole recombination rate of PCN‐222.

Time‐resolved photoluminescence spectroscopy (TRPL) was further employed to dynamically reveal interfacial electron transfer during light reaction. As depicted in Figure 4d, the TRPL data was fitted with a three‐exponential decay function, which can be expressed by the following formula

(1) $$\begin{array}{ccc} \tau_{\mathrm{avg}} & = & A+B_{1\;}\times \exp\left(-\frac{i}{\tau_{1}}\right)+B_{2\;}\times \exp\left(-\frac{i}{\tau_{2}}\right)+B_{3\;}\\ & & \times \;\exp\left(-\frac{i}{\tau_{3}}\right) \end{array}$$

The results of the fluorescence lifetime measurements revealed that, upon excitation with 405 nm fluorescence, the photo‐generated electrons of PCN‐222 were quenched after 0.39 ns, while those of the biohybrid were quenched significantly faster, at 0.19 ns (as detailed in Table S4, Supporting Information). This shorter fluorescence lifetime strongly provides compelling evidence that the photo‐generated electrons generated by PCN‐222 were swiftly transferred to *E. coli*. This observation aligns seamlessly with prior research in this domain.^[^ ^61^ ^]^ To delve deeper into the interfacial electron transfer dynamics, we introduced varying concentrations of Cr^6+^ as an electron transfer eliminator, given its strong competition with the cell for electrons.^[^ ^62^ ^]^ The results, as illustrated in Figure 4e, unmistakably indicate a decrease in lysine production as the concentration of Cr^6+^ was increased. This compelling evidence strongly reinforces the notion that electron transfer at the material–cell interface is intricately linked to the light‐driven synthesis of lysine.

**Figure 5:**
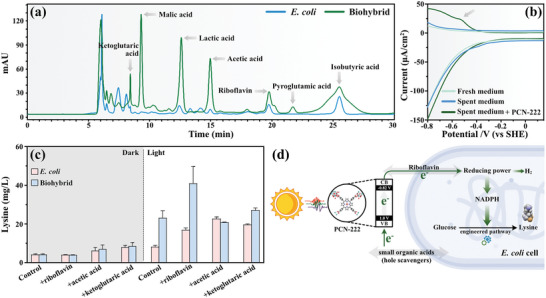
Analysis of released redox substances and proposed working mechanism of the biohybrid. a) HPLC analysis of the supernatant after the light reaction of the biohybrid. b) DPV measurements of fresh medium and the spent medium (supernatant collected from the light‐irradiated biohybrid after reaction) with or without PCN‐222 coated on carbon paper. c) Lysine production of *E. coli* and biohybrid after adding riboflavin and small molecule acids. d) Proposed mechanism for the transmembrane photoelectron delivery and their effect on intracellular biosynthesis pathways in the biohybrid.

Numerous electroactive bacteria have evolved specialized channels on their outer membrane to facilitate transmembrane electron transport.^[^ ^63^ ^]^ Despite the presence of a wide range of dehydrogenases and redox enzymes in the inner membrane, *E. coli* has traditionally been regarded as a non‐electroactive microorganism due to the absence of electron channels or conduits in outer membrane, which restricts the direct transfer of extracellular electrons into the cells.^[^ ^64^ ^]^ Recent studies have showcased the potential of specific endogenous redox substances in *E. coli*, such as quinones, riboflavin, pyrimidine derivatives, and hydroquinone derivatives, to serve as soluble electron transfer mediators.^[^ ^60^, ^65^, ^66^ ^]^ To assess the release of soluble substances by *E. coli* and the biohybrid, we collected the spent medium after 24 h of light exposure and subjected it to high‐performance liquid chromatography (HPLC) analysis. According to the data spectrum of HPLC, no small molecular acids and riboflavin were found in the control group or the material group in the dark (Figure S13, Supporting Information). However, after 24 h reaction under light, the secretion of various endogenous substances, encompassing small molecule acids and riboflavin, by both *E. coli* and the biohybrid (As depicted in **Figure** **5**a). Remarkably, the biohybrid group exhibited markedly higher peak areas for ketoglutaric, malic, lactic, and acetic acids, and notably, it uniquely secreted pyroglutamic acid. Specifically, the reduction potentials of ketoglutaric acid (−0.38 V) and acetic acid (−0.7 V), fall within the conduction band of PCN‐222 (−0.82 V), and NADP^+^/NADPH (−0.32 V). This intriguing alignment suggests that these small molecule acids may serve as mediators, actively facilitating the transfer of photogenerated electrons into cells, ultimately bolstering NADPH generation. In addition, riboflavin, which is an electron‐carrying cofactor in natural photosynthesis systems and has been reported to facilitate electron transfer in microbial photoelectrochemical systems,^[^ ^67^, ^68^, ^69^, ^70^ ^]^ was also found to be overproduced by the biohybrid (Figure 5a). This finding strongly suggests that riboflavin, too, may play a vital role in enhancing the transfer of photo‐generated electrons into the cells, further augmenting the overall electron transfer efficiency of the biohybrid system. Differential pulse voltammetry (DPV), which has peaks originating from electrochemically active substances from the cells at the electrode surface, was further employed to study the key component for electron mediation at the material–cell interface. As illustrated in Figure 5b, it can be found that when the blank carbon paper is used as the working electrode, the test curve does not show a peak. After PCN‐222 is coated on the carbon paper electrode, it shows a peak near −0.5 V, which is due to the fluctuation caused by the oxidation reaction, referred to as the oxidation peak, which is more negative than the thermodynamic potential of NADP^+^/NADPH (*E*_0_ = −0.32 V vs SHE) and the valence band potential of PCN‐222 (1.0 V).^[^ ^63^ ^]^ This compelling evidence solidifies the notion that riboflavin, released by the biohybrid throughout the reaction, plays a pivotal role in the electron transfer process.

The crucial role of riboflavin and small organic acids in the electron transfer process for lysine production was subsequently confirmed. As depicted in Figure S14 (Supporting Information), the introduction of riboflavin to the *E. coli* group (as shown in Figure 5c) resulted in a significant 105% increase in lysine production compared to the pure *E. coli* under light conditions. According to this result, riboflavin does indeed function as a photosensitizer to some extent. The biohybrid group, in the absence of added riboflavin, exhibited a remarkable 181% increase in lysine production compared to the pure *E. coli* group. Moreover, an impressive 396% surge in lysine production was observed in comparison to the pure *E. coli* group. This finding highlights the crucial role of riboflavin in facilitating NADPH production through electron transfer. Thus, it can be concluded that riboflavin acts primarily as a mediator in this system, rather than solely functioning as a photosensitizer. Additionally, the integration of small organic acids into the biohybrid resulted in noteworthy improvements in the dark group, confirming their potential as valuable carbon sources that fulfill the energy requirements of *E. coli* cells. Intriguingly, the augmented lysine production in the biohybrid containing small organic acids in the light group hints at an additional role for these acids as sacrificial hole scavengers in electron transfer.

Based on the results, we have proposed a mechanism for the light‐driven production of lysine by the PCN‐222‐*E. coli* hybrid system, as illustrated in Figure 5d. The biohybrid was formed through self‐assembly of the surface positively charged PCN‐222 and negatively charged *E. coli*, facilitated by electrostatic interactions. Under light exposure, PCN‐222, with its outstanding light‐capture performance, generates a substantial quantity of photo‐generated electrons that are efficiently transferred into *E. coli* cell. Regarding electron transfer, the photogenerated electrons produced by PCN‐222 can stimulate the secretion of endogenous riboflavin. This riboflavin acts as an electron mediator, aiding in the transmembrane transport of electrons. At the same time, the small organic acids could serve as the hole scavengers to PCN‐222 to facilitate the electron transfer and migration. As a result, the photo‐generated electrons obtained by *E. coli* leads to an elevation in the intracellular NADPH level, resulting in excess reducing power being converted into lysine, thus achieving efficient light‐driven lysine synthesis.

## 3 Conclusion

In this study, we successfully developed a self‐assembling hybrid system combining PCN‐222 and *E. coli* through electrostatic interaction forces and continuous covalent interaction. The light‐irradiated biohybrid system could efficiently produce H_2_ with a quantum efficiency of 0.75% under full‐spectrum irradiation. And the elevated intracellular reducing power NADPH was unequivocally demonstrated. Additionally, we investigated the performance of light‐driven lysine production using genetically engineered *E. coli*. Under optimized conditions, the biohybrid system achieved an impressive lysine production of 18.25 mg L^−1^, surpassing pure bacterial production by an astounding 332%. Furthermore, our investigation into electron transfer mechanisms at the material–cell interface yielded intriguing insights. PCN‐222 adeptly harnessed light energy, facilitating the seamless transfer of photo‐generated electrons into *E. coli* cells. This interfacial energy transfer process was orchestrated by the involvement of riboflavin, with additional support from secreted small organic acids acting as effective hole scavengers for PCN‐222. This study not only underscores the proficiency of metal porphyrin–organic frameworks as formidable photoelectron donors for vital non‐photosynthetic industrial microbial cell factories, enabling the light‐driven synthesis of fuels and valuable chemicals, but also lays a solid foundation for future endeavors aimed at enhancing the efficiency of *E. coli*‐based biohybrid systems.

## 4 Experimental Section

### Synthesis and Characterization of PCN‐222

To prepare the PCN‐222 material, ZrCl_4_ (0.515 mmol) and C_6_H_5_COOH (33.16 mmol) were dissolved in 24 mL of *N*,*N*‐diethylformamide and sonicated for 1 h to obtain a uniform slurry. The mixture was then heated at 90 °C for 2 h. Next, TCPP (0.075 mmol) was added to the heated solution, sonicated for 20 min, and heated at 120 °C for 48 h. After cooling to room temperature, the resulting violet‐colored plate‐like crystals were filtered and dried at 70 °C for 12 h in a vacuum oven.^[^ ^38^ ^]^ The micro morphology of PCN‐222 was characterized using an SEM (ZEISS EVO 18, Germany), while the material structure was analyzed by XRD (Rigaku D/MAX 2500 V, Japan). FTIR (Thermo Scientific iN10, USA) was used for material testing, and the elemental composition of PCN‐222 was analyzed using XPS (Thermo Scientific Nexsa, USA). The absorbance of PCN‐222 was measured in the range of 200–800 nm using a UV–vis spectrophotometer (Shimadzu UV‐3600, Japan).

### Construction and Characterization of Biohybrid System

*E. coli* BL21(DE3) (Source of original strain: Yu Tao research group, research center of synthetic biochemistry, Shenzhen Institute of advanced technology, Chinese Academy of Sciences) was cultured in fresh LB medium with chloramphenicol (25 mg mL^−1^). The 37 °C incubated cell culture at OD_600_ = 0.6 was collected, arabinose (1 m) and different concentrations of PCN‐222 were added to prepare the mixtures according to the experimental setting. The mixture was induced in a 16 °C shaker for 24 h, then centrifuged and washed twice with sterile phosphate‐buffered saline (PBS). The LB medium was replaced with M9 medium (Table S1, Supporting Information). Subsequently, the culture medium was divided into 20 mL aliquots and transferred into sterilized photoreaction bottles with a total volume of 50 mL. The photoreaction bottles were securely sealed and positioned into the Multi‐Channel (automatic temperature control) Photochemical Reaction System (PCX‐50C, Beijing Perfectlight, China) with a light intensity of 38.93 mW cm^−2^ and a temperature of 37 °C. The surface morphology of the mixture was photographed using a desktop field emission SEM (Pharos, Phenom, China), and the Zr, C, N, and O elements in the sample were scanned by energy spectrum (15 kV). The experimental samples for SEM were prepared using the alcohol gradient dehydration method. The surface morphology of the biohybrid was observed by AFM. The surface potential of the experimental samples was analyzed using a Zetasizer pro (Blue/Red) potentiometer (Malvern, UK). FTIR (Thermo Scientific iN10, USA) was used for surface functional groups of bacteria, materials, and biohybrid testing. The microplate reader (Tecan Infinite M200 NanoQuant absorbance microplate reader, TECAN, Switzerland) was used for the absorbance of bacteria, materials, and biohybrid testing.

### The living and Dead Staining Experiment of Bacteria

A stock solution of PI was prepared by diluting it to a concentration of 0.1 mg L^−1^ (1.5 × 10^−7^ mol) using pure water. Similarly, FITC was diluted to a concentration of 1.5 × 10^−7^ mol using pure water. The diluted PI and FITC solutions were thoroughly mixed to obtain a homogeneous mixed dye solution. 2 mL microcentrifuge tube was taken and it was filled with 400 µL OD_600_ = 2, and 100 µL mixed dye, wrapped the EP tube with tinfoil, and incubated at room temperature in darkness for 5 min. The stained bacteria were collected by centrifugation at 10 000 rpm for 3 min and washed twice with PBS. After the washing steps, the bacteria were resuspended in 100 µL of PBS. A drop of the bacterial suspension was placed on a glass slide and covered with a coverslip. Observation of bacterial viability was performed using a confocal microscope (AX confocal laser scanning micros, Nikon, Japan).

### Measurement of H_2_ and Intracellular Energy Forms

*E. coli* MG1655 (wild‐type) (Source of original strain: Professor Yi Xiao's research group, research center of synthetic biochemistry, Shenzhen Institute of advanced technology, Chinese Academy of Sciences) was activated by culturing in fresh LB medium at 37 °C for 7 h, followed by centrifugation and washing twice with sterile PBS. Next, the *E. coli* was cultured in BC medium (Table S2, Supporting Information) and mixed with PCN‐222 to prepare the biohybrid samples at the ratio of 3 mg/20 mL. The biohybrid samples were aced into an anaerobic tank in a 37 °C shaker overnight, then centrifuged and cleaned twice with sterilized PBS, and placed in SBC medium (Table S3, Supporting Information). Aliquots of 20 mL culture medium were transferred to 50 mL photo‐reaction bottles filled with nitrogen. The sealed photo‐reaction bottles were placed into the multi‐channel (automatic temperature control) photocatalytic reaction system (PCX50C Discover, PefectLight, China) with a light intensity of 38.93 mW cm^−2^ and a temperature of 37 °C. The reaction lasted for 4 h, with hourly sampling conducted. The H_2_ content was measured using a gas chromatograph (GC9790 II, Fuli, China), while the intracellular NADPH/NADP^+^ was measured with the NADP^+^/NADPH assay kit with WST‐8 (S0179, Beyotime Biotechnology). The absorbance was measured using a 96‐well plate reader (Tecan Safire Abs, Switzerland), and the concentrations of the individual compounds were calculated using standard curves.

### Measurement of Lysine Production

*E. coli* BL21(DE3) was genetically modified to overexpress the lysine synthesis pathway (Figure S15, Supporting Information). The bacterial strain *E. coli* BL21 (purchased from Jiangsu Cowin Biotech Co., Ltd) was used as the host cell for gene transformation. Fragments of *asd, ppc*, *LysC^T344M^*, *DapA^H56K^*, and *DapB* were amplified from the *E. coli* BL21(DE3) genome, while the *pos5* fragment was amplified from the *Saccharomyces cerevisiae* genome (the relevant agarose gel electrophoresis is shown in Figure S16 (Supporting Information), source of original strain: yeast BY4742 from Professor Yu Tao's research group, research center of synthetic biochemistry, Shenzhen Institute of advanced technology, Chinese Academy of Sciences). The relevant plasmid was pTht006. The sequences were synthesized by Sangon Biotech with codon optimization. The relevant promoter and primers sequences are labeled in Table S6 (Supporting Information). The recombinant plasmids were constructed by Gibson assembly. The induced *E. coli* BL21 was centrifuged from LB and washed, then placed in M9 medium and divided into 20 mL portions, which were transferred into 50 mL photo‐reaction bottles to initiate the photo reaction with a light intensity of 38.93 mW cm^−2^ at 37 °C. To detect lysine, 1 mL of bacterial solution at different time intervals was taken during the reaction, centrifuged at 10 000 rpm for 5 min, and 100 µL of supernatant was taken for freeze‐drying. The sample was dissolved again with 1 mL of 60% methanol solution, filtered through a 0.22 µm organic membrane filter, and added to a sample bottle for detection. The extracts were analyzed using a UPLC‐QqQ‐MS/MS (LCMS, 1260‐Ultivo, Agilent Technologies, USA) system equipped with an electrospray ionization source.

### Experiment of the System Durability

The preliminary steps of this experiment were consistent with the experimental steps described in “Measurement of lysine production.” Within 3 days, after every 24 h reaction, 1 mL of the solution was taken and it was centrifuged. The supernatant was taken and it was freeze dried. Then 60% methanol was used for remelting. After sampling, pure *E. coli* and biohybrid were centrifuged from the used M9 medium, cleaned twice with PBS, and washed twice with sterile PBS. Then they were resuspended separately in fresh M9 medium.

### Photoelectrochemical Measurement and Electron Scavenger Study

To prepare the working electrode, carbon paper was cut into rectangular pieces of size 2 × 1 cm. 5 mg of PCN‐222 were dissolved in a mixture of isopropanol and Nafion, applied evenly after ultrasound. For preparing the biohybrid samples, the culture medium of PCN‐222‐*E. coli* was centrifuged, the supernatant was discarded, and the cells were rinsed once with PBS. The cells were then dissolved in a mixture of isopropanol and Nafion, and the liquid was applied evenly onto the carbon paper. The working electrode, Ag/AgCl reference electrode, and Pt wire electrode were installed in a 50 mL quartz electrolytic cell with 30 mL of 0.5 m Na_2_SO_4_ electrolyte solution and LED white light with a light intensity of 38.93 mW cm^−2^. Photocurrent and electrochemical impedance spectroscopy tests were performed on the electrochemical workstation (CHI760E, CH Instruments, Shanghai). Different concentrations of potassium dichromate (0, 0.1, 0.3, and 0.5 mm) were added into four groups of reaction bottles at the beginning of the light reaction, with Cr^6+^ as an electron transfer eliminator, and lysine output was detected in each reaction bottle after 24 h of light reaction.

### Measurement of the Released Soluble Substances for Electron Transfer

To investigate the released soluble substances, the spent medium of pure bacteria and biohybrid was collected after 24 h of light exposure. HPLC (1260 Infinity II, Agilent Technologies, USA) was used to determine the types of metabolites produced by the reaction. The Aminex HPX‐87H model column (7.8 × 300 mm) was selected, with 5 mm sulfuric acid as the mobile phase. The column oven temperature was set to 60 °C, the RID detector was set to 55 °C, and the flow rate was 0.6 mL min^−1^. The key components of electron mediation at the interface were further determined using DPV of the electrochemical workstation. The reactor for further study was formed using the culture medium after the reaction and the blank working electrode (pure carbon paper) or PCN‐222 working electrode. The function verification experiment of riboflavin and small molecular acid was carried out by adding 20 µmol riboflavin, acetic acid, and ketoglutaric acid to the system respectively, with a light intensity of 38.93 mW cm^−2^, a temperature of 37 °C, and rotating speed of 220 rpm. The lysine output was detected in each reaction bottle after 24 h of light reaction.

### Detection of Electron Transfer at the Interface of Biohybrid System

The recombination efficiency of electrons and holes between PCN‐222 and the biohybrid system was compared by photoluminescence (PL). The PL measurements were performed in the emission mode, employing a 5 nm slit width. A specific excitation wavelength of 413 nm was employed, while the emission wavelength range spanned from 450 to 600 nm. The fluorescence lifetime of PCN‐222 and the biohybrid system was compared by TRPL. The instrument mode utilized was TCSPC‐time life mode, with an excitation wavelength of 405 nm. The measurement range encompassed 10 ns (100 MHz). A delta diode light source was employed to generate the excitation light. To ensure accurate measurements, a 450 nm filter was carefully positioned within the emission chamber of the sample to prevent any excitation wavelength light from interfering with the detector. Subsequently, the acquired data were subjected to analysis and fitting using the “Eztime” software, enabling a thorough characterization of the fluorescence lifetime dynamics exhibited by both PCN‐222 and the biohybrid system.

### Determination of Apparent Quantum Efficiency

The calculation methods of apparent quantum yield (AQE) in the literature were mostly applicable to monochromatic light or single wavelength,^[^ ^10^ ^]^ such as

(2) $$\mathrm{AQE}\;=\frac{N_{H_{2}}}{N_{p}}\;=\frac{2\;\times \;\mathrm{Additional}\;\mathrm{generated}\;H_{2}\;\mathrm{number}\;\mathrm{of}\;\mathrm{molecules}}{\mathrm{Number}\;\mathrm{of}\;\mathrm{incident}\;\mathrm{photons}}\;\times 100\%\;=\;\frac{2\;\times \;N_{a\;}\times \;M_{H_{2}}}{\frac{PSt\lambda }{hc}}\;\times 100\%$$

where *N*_a_ is the amount of hydrogen material, $M_{H_{2}}$ is the molar mass of hydrogen production of biohybrid system, the factor of 2 accounts for 2 e^−^ to convert to hydrogen. *N*_p_ is the total incident photons. *P*, *S*, *t*, *λ*, *h*, and *c* are the power of the light at a certain wavelength (mW cm^−2^), active area (7.07 cm^2^), total reaction time (14 400 s), incident light wavelength (nm), Planck constant (6.62 × 10^−34^ J s), and speed of light (3 × 10^8^ m s^−1^), respectively.

In this paper, white light was used to irradiate the hybrid system instead of monochromatic light. For a wide spectrum, the number of photons at each wavelength needs to be calculated separately and then summed because the energy of photons at each wavelength was different. So, the calculation method of *N_p_* should be changed to

(3) $$N_{p}=\frac{St}{hc}\;\Sigma P\lambda$$

Applying the data to the *N_p_* formula, the value of *N_p_* is 2.035 × 10^22^. It is known that the $M_{H_{2}}$ under light is 126.7615 µMol, which is substituted into the above formula to obtain

(4) $$\begin{array}{ccc} \mathrm{AQE} & = & \frac{2\;\times \;126.7615\;\times \;10^{-6}\;\times \;6.00\;\times \;10^{23}}{2.035\;\times \;10^{22}}\;\times 100\%\\ & = & 0.75\% \end{array}$$

Therefore, according to the new formula, the AQE of PCN‐222‐*E. coli* system in the hydrogen production of optical drive was 0.75%.

### Statistical Analysis

Unless otherwise indicated, statistical analysis was performed in Graphpad Prism 8. The samples chosen for analysis were derived from at least three biologically independent experiments. Data were presented as graphs or in‐text showing the mean values ± SD as appropriate.

## Conflict of Interest

The authors declare no conflict of interest.

## Author Contributions

J.L. and J.S. contributed equally to this work. J.L.: investigation, data curation, formal analysis, methodology, and writing—original draft. J.S.: investigation, data curation, and methodology. T.H.: resources. H.T.: supervision and resources. C.Z.: supervision and resources. K.X.: project administration, writing, review, and editing. Y.H.: supervision and resources. B.W.: supervision, funding acquisition, resources, writing—review, and editing.

## Supporting information

Supporting Information

## Data Availability

The data that support the findings of this study are available from the corresponding author upon reasonable request.
