# Supplementary material for: A Self‐Assembled MOF‐Escherichia Coli Hybrid System for Light‐Driven Fuels and Valuable Chemicals Synthesis
Source: Adv Sci (Weinh). 2024 Apr 25;11(25):2308597. doi: 10.1002/advs.202308597 (PMC11220693; doi:10.1002/advs.202308597)
Supplement: Supplementary file 1 — Supporting Information [file ADVS-11-2308597-s001.pdf]

ADVANCED SCIENCE
Open Access

Supporting Information

for *Adv. Sci.*, DOI 10.1002/advs.202308597

A Self-Assembled MOF-*Escherichia Coli* Hybrid System for Light-Driven Fuels and Valuable Chemicals Synthesis

Jialu Li, Junfeng Shen, Tianfeng Hou, Hongting Tang, Cuiping Zeng, Kemeng Xiao, Yanping Hou* and Bo Wang**

Supporting Information

A self-assembled MOF-*Escherichia coli* hybrid system for light-driven fuels and valuable chemicals synthesis

Jialu Li, Junfeng Shen, Tianfeng Hou, Hongting Tang, Cuiping Zeng, Kemeng Xiao, Yanping Hou* and Bo Wang**

RESULTS

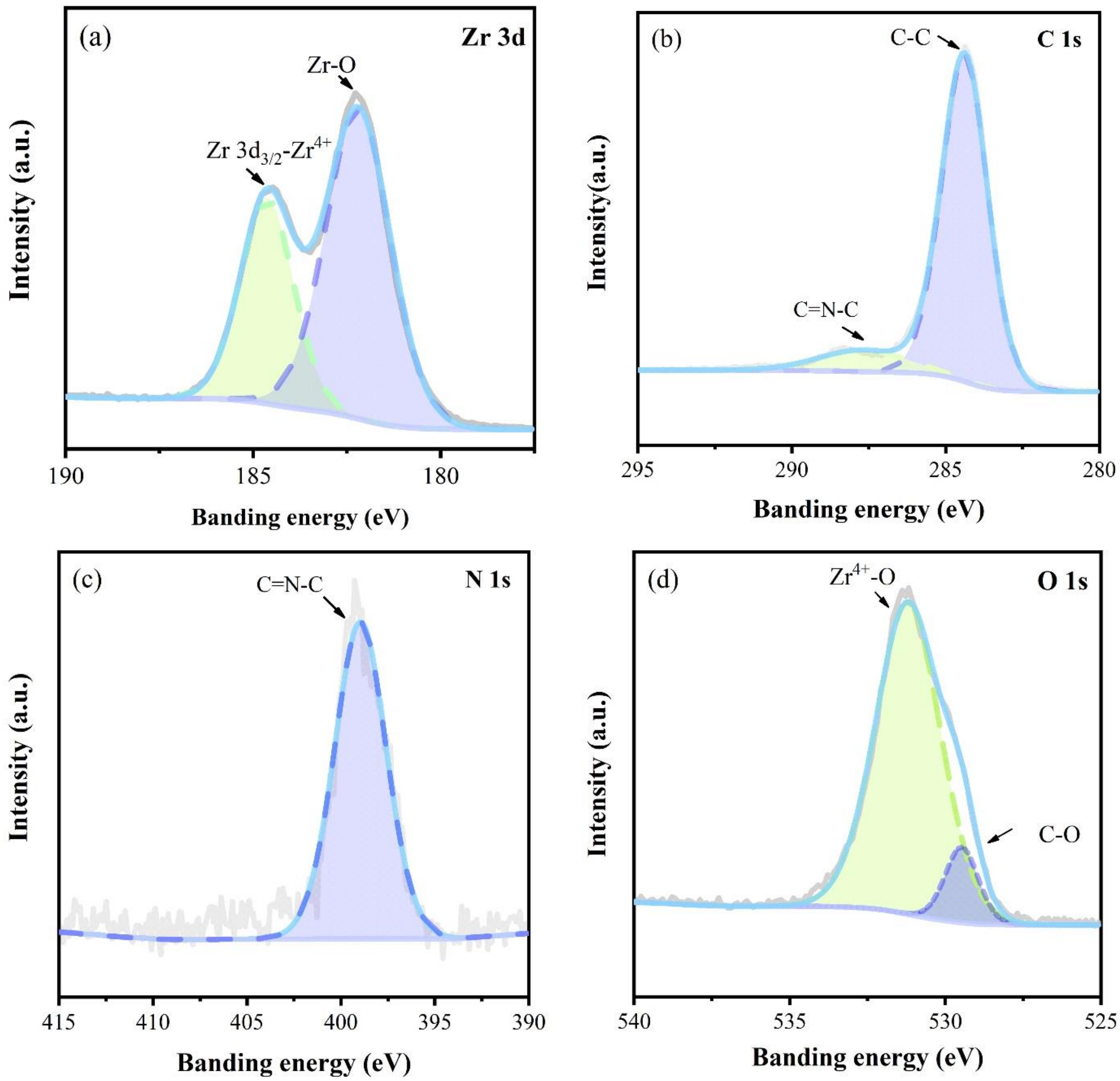

Figure S1. High resolution XPS survey spectra for (a) Zr 3d, (b) C 1s, (c) N 1s and (d) O 1s.

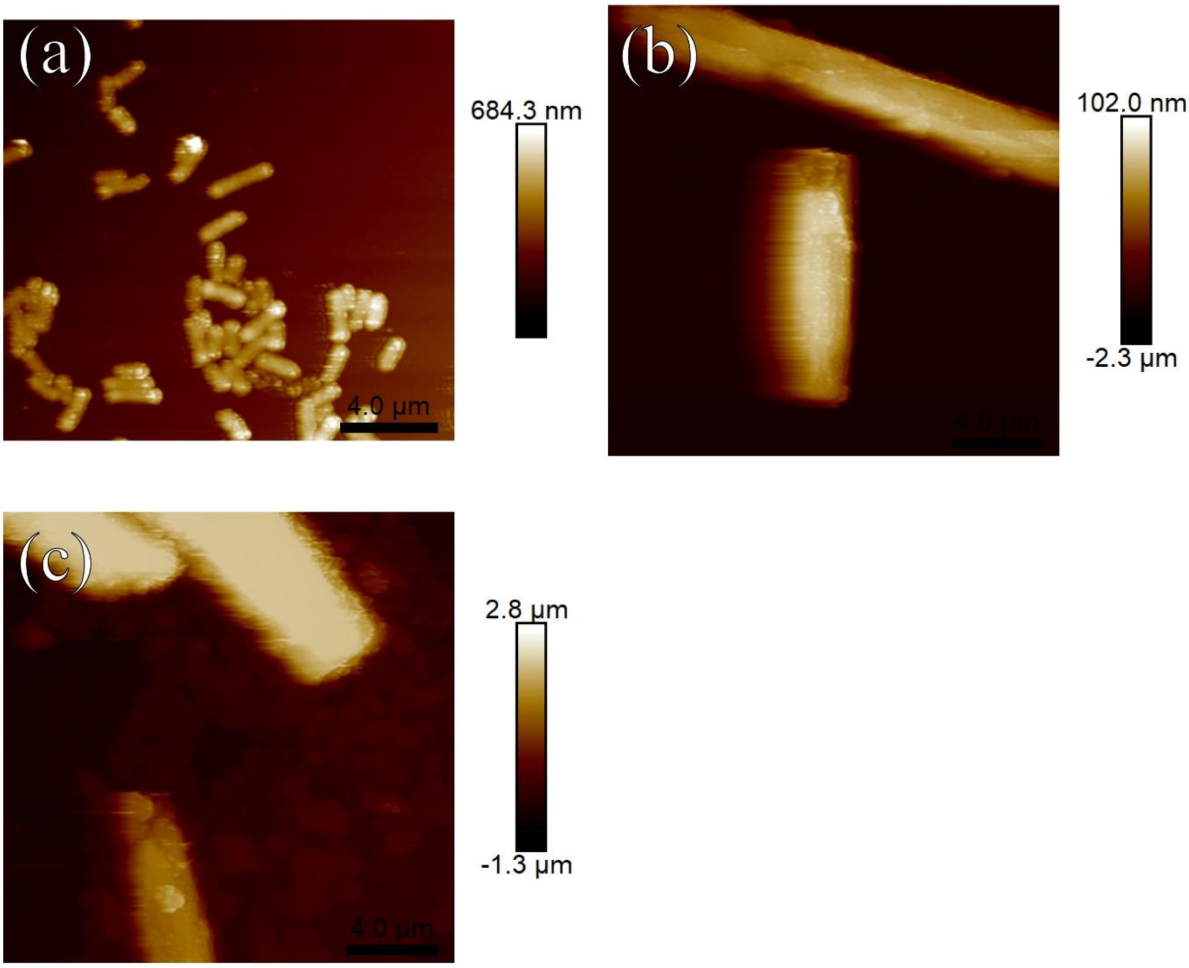

Figure S2. AFM images showing both the topography and height of (a) *E. coli* (b) PCN-222 and (c) biohybrid.

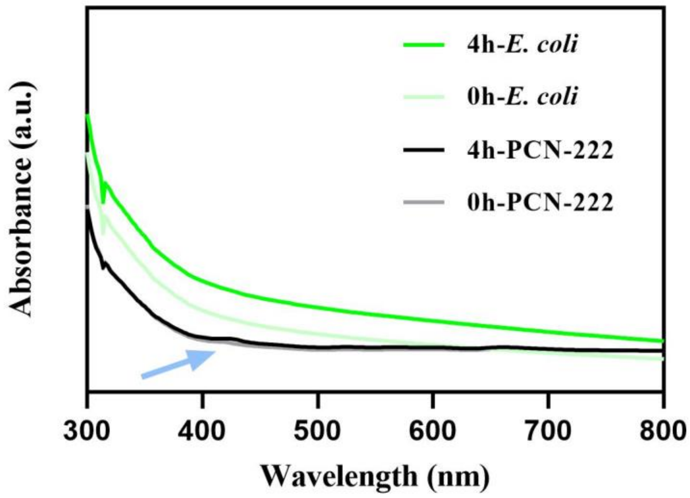

Figure S3. Absorbance spectra of *E. coli* and PCN-222 within 4 h.

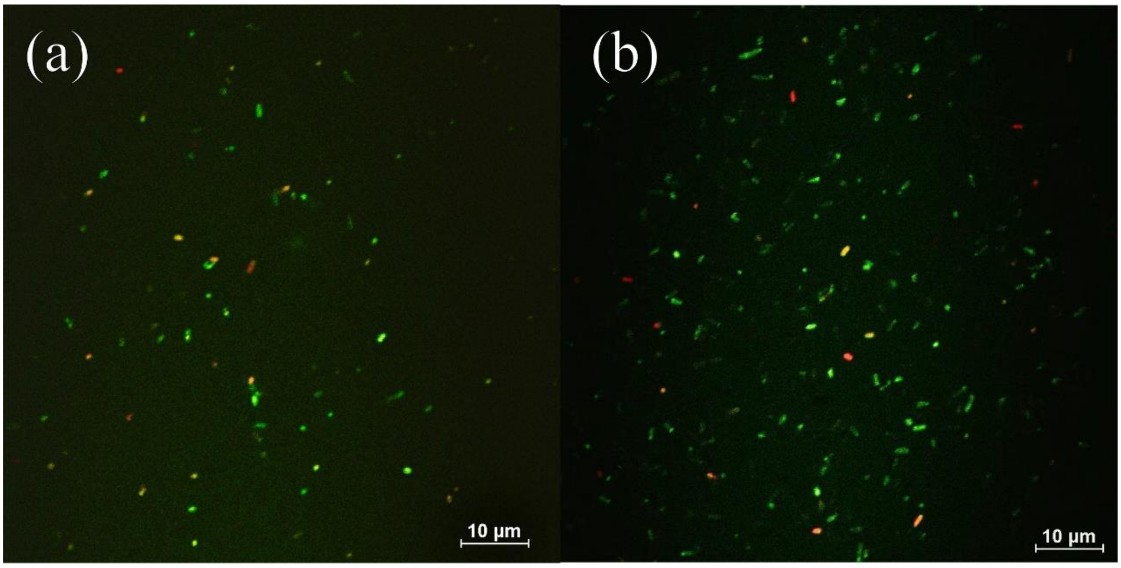

Figure S4. (a-b) Staining fluorescence image of engineered *E. coli* before and after 24 h dark reaction.

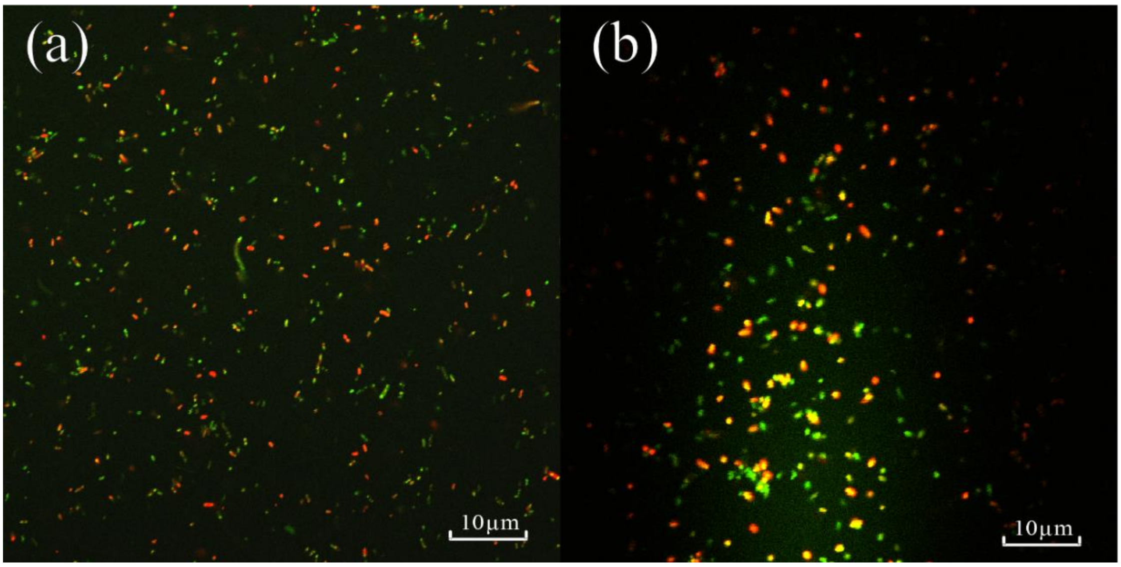

Figure S5. Staining fluorescence image of (a) *E. coli* and (b) biohybrid before and after 24 h light reaction.

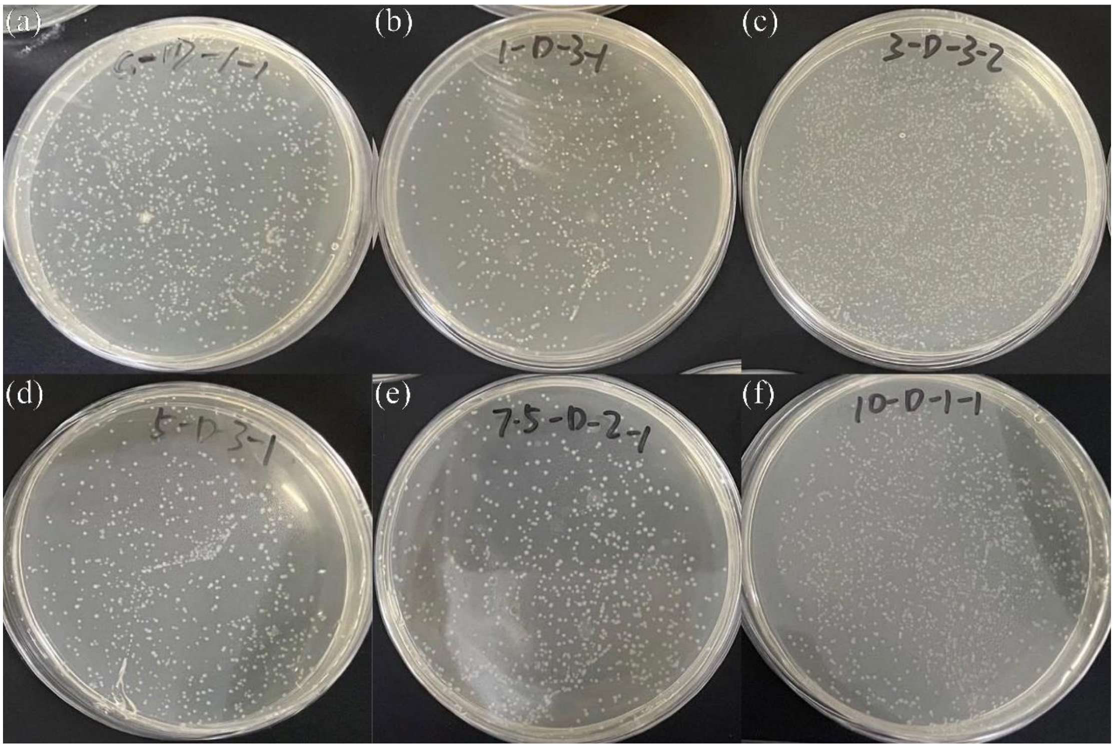

Figure S6. Plating experiments after dark reaction for (a) *E. coli*; (b) PCN-222 (1 mg)-*E. coli*; (c) PCN-222 (3 mg)-*E. coli*; (d) PCN-222 (5 mg)-*E. coli*; (e) PCN-222 (7.5 mg)-*E. coli* and (f) PCN-222 (10 mg)-*E. coli*. (*E. coli* strain: BL21(DE3))

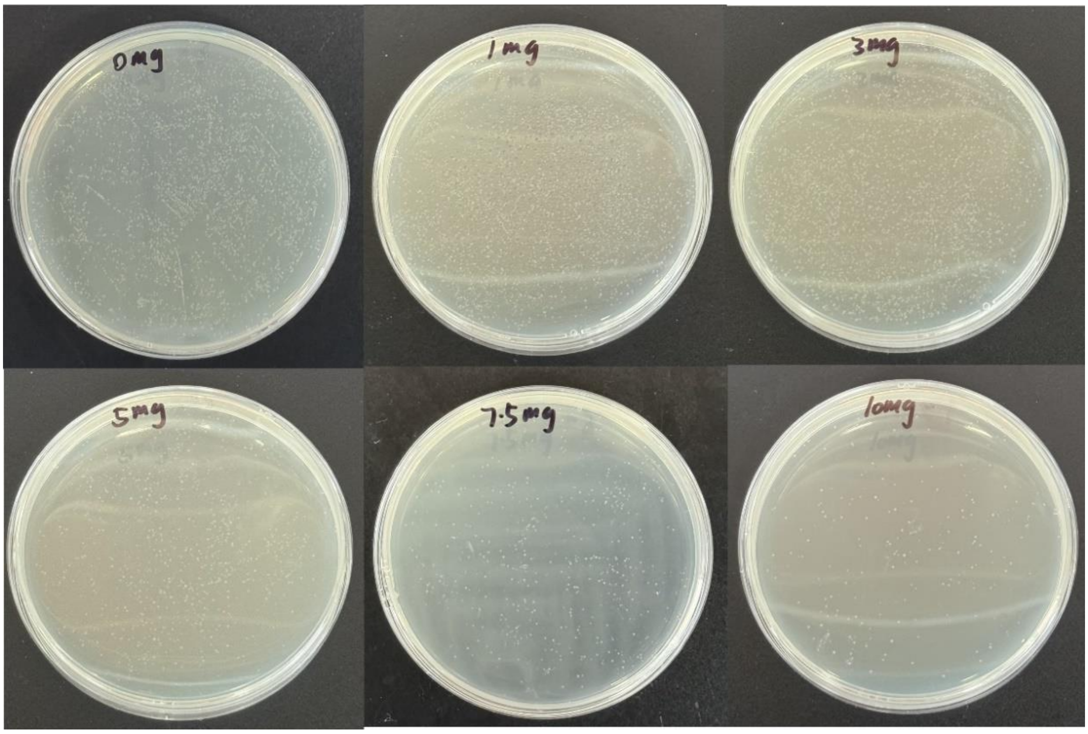

Figure S7. Plating experiments after light reaction for (a) *E. coli*; (b) PCN-222 (1 mg)-*E. coli*; (c) PCN-222 (3 mg)-*E. coli*; (d) PCN-222 (5 mg)-*E. coli*; (e) PCN-222 (7.5 mg)-*E. coli* and (f) PCN-222 (10 mg)-*E. coli*. *E. coli* strain: BL21(DE3)

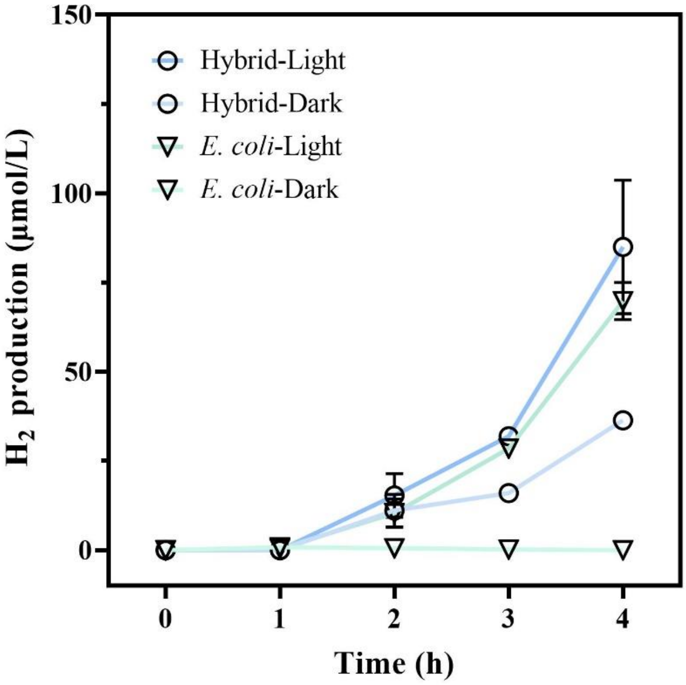

Figure S8. H_2 evolution of pure engineered *E. coli* and biohybrid in the dark versus light (38.93 mW/cm^2)

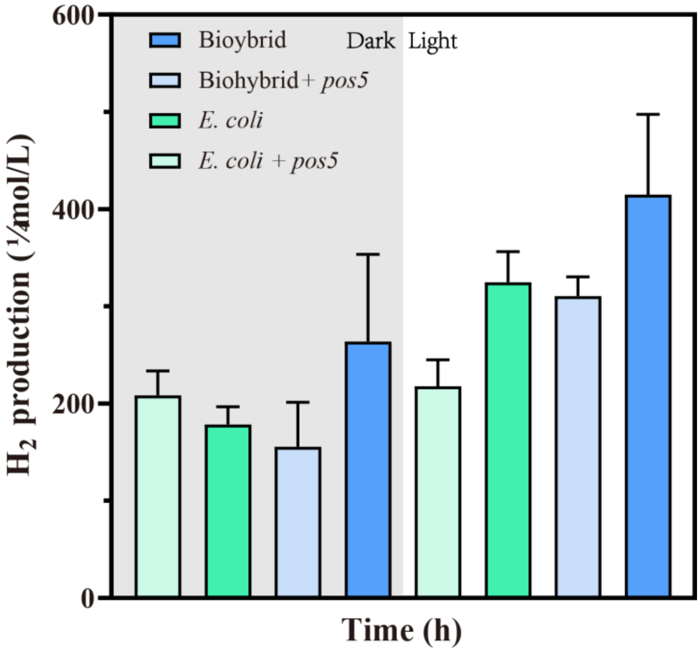

Figure S9. Experiment on whether *pos5* will affect the hydrogen production ability of engineered *E. coli* and biohybrid in the dark versus light after 24 h reaction (*E. coli* (without *pos*5); *E. coli* + *pos5* (with *pos*5), Biohybrid (without *pos*5) and Biohybrid + *pos5*(with *pos5*); (38.93 mW/cm^2).

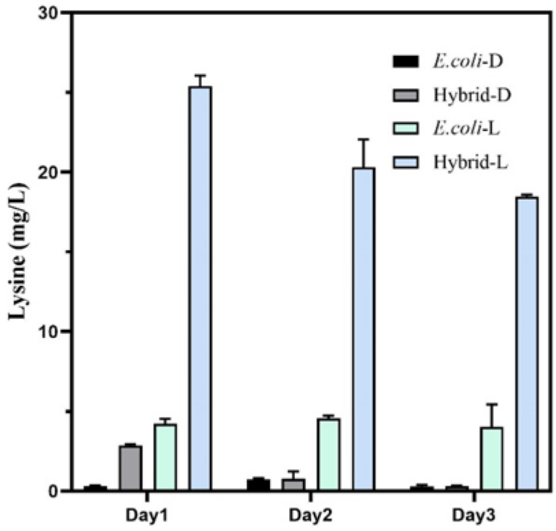

Figure S10. The durability of pure engineered *E. coli* and biohybrid in the dark versus light (38.93mW/cm^2).

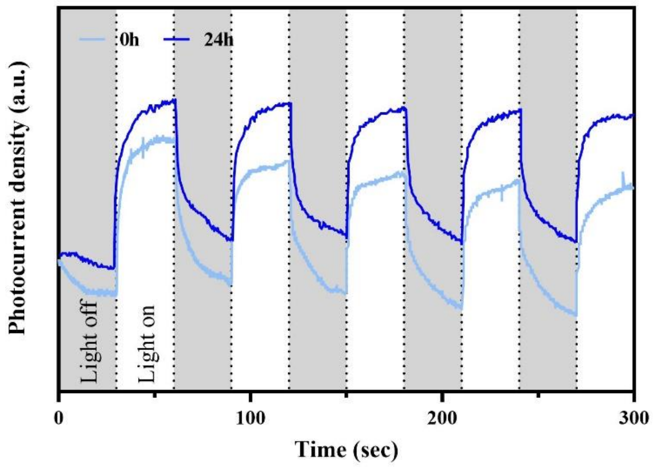

Figure S11. Photocurrent response of biohybrid before and after 24 h light reaction. (38.93 mW/cm^2).

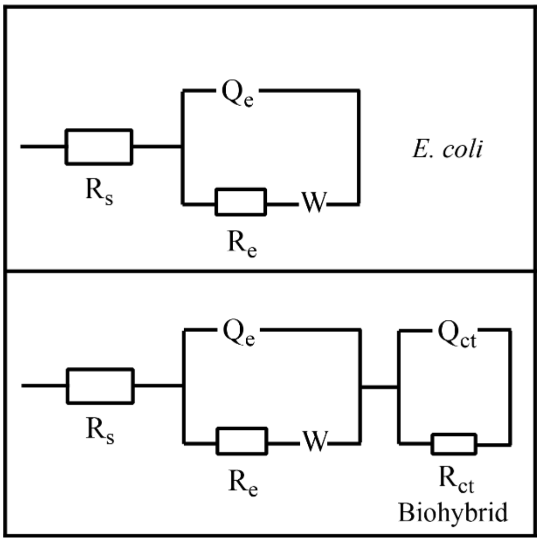

Figure S12. Equivalent circuit diagram of pure *E. coli* and biohybrid system. Parameters for equivalent circuit diagram: R_s, solution resistance; R_e, bacterial resistance; R_{ct}, PCN-222 resistance; Q_e, capacitance of the biofilm; Q_{ct}, capacitance of the electric double layer; W, Warburg impedance. (38.93 mW/cm^2)..

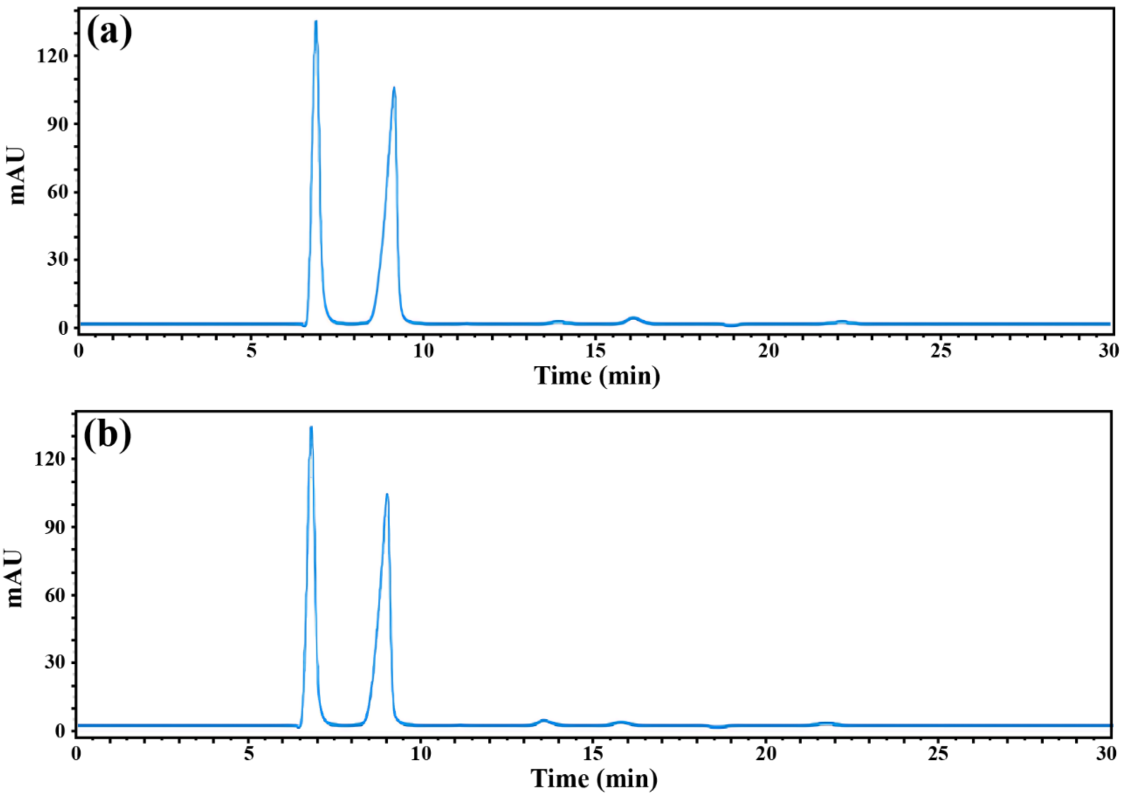

Figure S13. HPLC analysis of the supernatant after the dark reaction of (a) *E. coli* and (b) biohybrid.

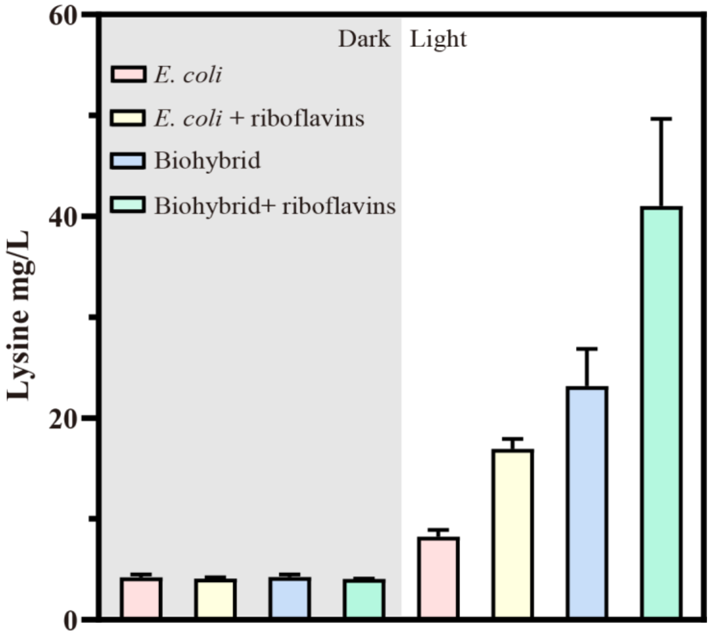

Figure S14. Lysine production of engineered *E. coli* with or without adding riboflavin and biohybrid with or without riboflavin. (38.93 mW/cm^2).

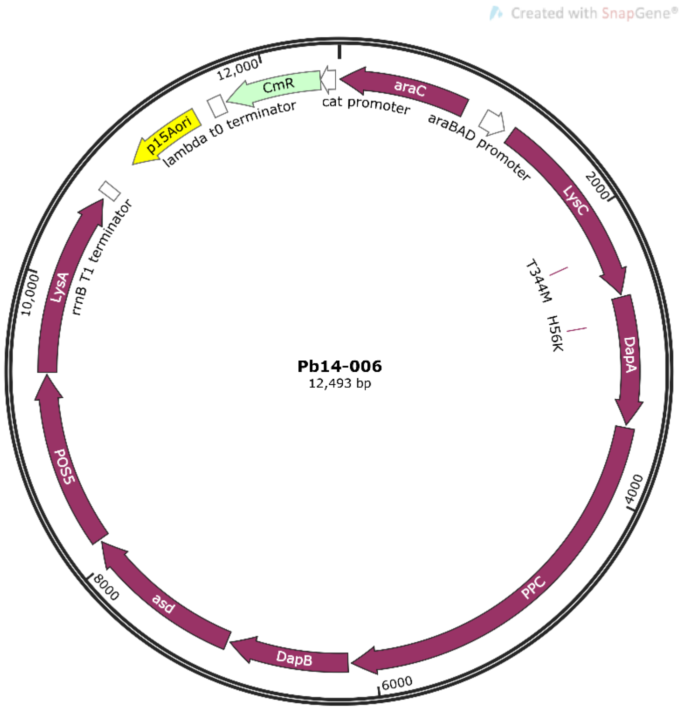

Figure S15. Co-expression of lysine synthetic pathway.

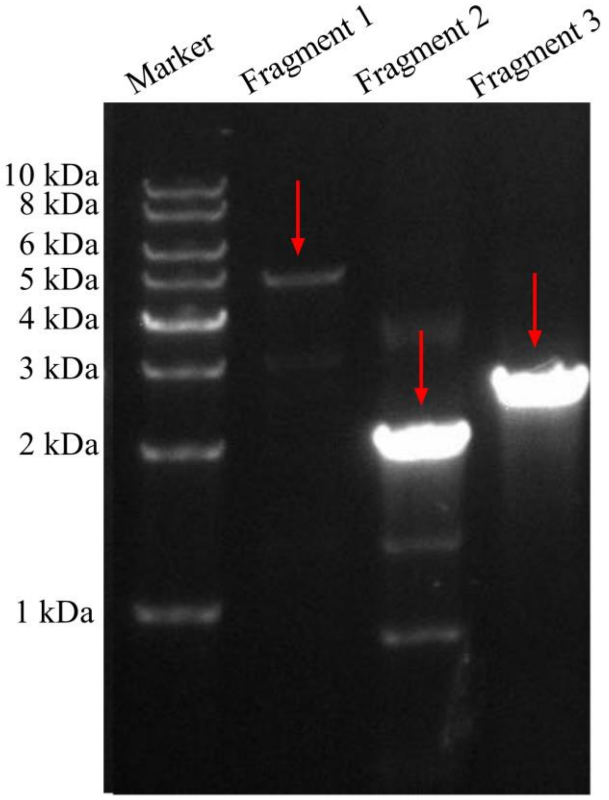

Figure S16. Electrophoretic experiment for verifying gene fragments insertion.

(Fragment1: *lysC*^{T344M}, *dapA*^{H56K}, *ppc*; Fragment2: *dapB*, *asd*; Fragment3: *pos5*, *lysA*)

Table S1. Comparison of quantum efficiency of correlated semi-artificial photosynthetic systems

Catalytic module	Photosensitizer	Product	AQE	Reference
E. coli	PCN-222	H_2	0.75% (under white light)	This work
M. barkeri	CdS	CO_2/CH_4	0.34% (under 395 nm LED light)	[1]
T. denitrificans	CdS	NO_3/NO	2.0% (under 395 nm LED light)	[2]
S. cerevisiae	InP	Hexose/ shikimic acid	0.85% (under the cold white light)	[3]
M. thermoacetica	$Au_{22}(SG)_{18}$	CO_2	2.86% (simulated sunlight)	[4]
E. coli	$AglnS_2/In_2S_3$	H_2	3.3% (under the irradiation of 720 nm xenon lamp)	[5]
[NiFe]-hydrogenase	Ag	H_2	2.3% (under 500 nm LED light)	[6]
[FeFe]-hydrogenase	Eosin Y	H_2	13.72% (under 460 nm LED light)	[7]
Light-sensitive protein	Photosensitizer Protein (PSP)	CO_2/CO	2.6% (under the irradiation of 400 - 450 nm xenon lamp)	[8]

Table S2. Related resistance parameters of *E. coli* and biohybrid systems

Name	Parameters	Dark	Light
E. coli	R_e (Ω/cm^2)	3968	3803
PCN-222-*E. coli*	R_e (Ω/cm^2)	1244	1089
	R_{ct} (Ω/cm^2)	2.65	3.74

Table S3. Multi-exponential fitting results of TRPL profiles

	A	B1	τ_1 (bins)	B2	τ_2 (bins)	B3	τ_3 (bins)	τ_{avg}(ns)
PCN-222	0	22.31	14.89	49.5	14.85	27.19	14.81	0.39
PCN-222 -*E. coli*	0	49.24	7.32	26.6	7.91	23.41	6.83	0.19

E. coli strain: BL21(DE3)

Table S4. Composition of 5 × M9 medium

Chemicals	Concentration ($g \cdot L^{-1}$)
Na_2HPO_4	30
KH_2PO_4	15
NH_4Cl	5
NaCl	2.5

When making 1 × M9, dilute 5 × M9 to 1 × M9 with sterile water and add the following sterile solutions: 1 mL of 1M $MgSO_4 \cdot 7H_2O$, 10 mL of 20% glucose (0.2%)

Table S5. Composition of BC Medium

Chemicals	Concentration ($g \cdot L^{-1}$)
$(NH_4)_2HPO_4$	10
K_2SO_4	2
NaCl	0.3
$MgSO_4 \cdot 7H_2O$ (filter)	0.2
$FeSO_4 \cdot 7H_2O$	0.004
$ZnSO_4 \cdot 7H_2O$	0.0009
$CuSO_4$	0.0004
$MnSO_4$	0.0002
$CaCl_2 \cdot 2H_2O$	0.0008
$Na_2B_4O_7 \cdot 10H_2O$	0.00009
$Na_2SeO_3 \cdot 5H_2O$	0.0006
$(NH_4)_6Mo_7O_{24}$	0.0004
$Ni(NH_4)_2(SO_4)_2$	0.0009
Adjust pH to 7.0	
Supplement Glucose (30 mM)	

Table S6. Composition of SBC Medium

Chemicals	Concentration ($g\cdot L^{-1}$)
$(NH_4)_2HPO_4$	10
K_2SO_4	2
NaCl	0.3
$MgSO_4\cdot 7H_2O$ (filter)	0.2
Adjust pH to 7.0	
Supplement Glucose (20 mM)	

Table S7. Promoter and primers used in this study

Name	Sequence
Arabad promoter	Aaagccatgacaaaaacgcgtaacaaaagtgtctataatcacggcagaaaagtccacattgattatttgcacggcgtcacactttgctatgccatagcatttttatccataagattagcggattctacctgacgctttttatcgcaactctctactgtttctccat
lysC	*LysC*-F: gatcttttaagaaggagatatacatATGTCTGAAATTGTTGTCTCCAAATTTGGCGG<br>*LysC*^{T344M}-R: AACATtttgctttcctccttcaTTACTCAAACAAATTACTATGCAGTTTTT
dapA	*dapA*-F: tgaaggaggaaagcaaaATGTTCACGGGAAGTATTGTCGCGAT<br>*dapA*^{H56K}-R: TTCGTTCATgatgttttcctcctactagTTACAGCAAACCGGCATGCTTAA
ppc	*ppc*-F: ctagtaggaggaaaacatcATGAACGAACAATATTCCGCATTGCGTAGTAAT<br>*ppc*-R: ttatttcctcctAGATCCtcaTTAGCCGGTATTACGCATACCTG
dapB	*dapB*-F: GCTAAtgaGGATCTaggaggaaataaccATGCATGATGCAAACATCCGCGTTGCCA<br>*dapB*-R: CATTTTTCATatgatctccctcctagatccTTACAAATTATTGAGATCAAGTACATCTCGC
asd	asd-F: ctaggagggagatcatATGAAAAATGTTGGTTTTATCGGCTGGC<br>asd-R: TcatctcataatcctcctagatccTTACGCCAGTTGACGAAGCATCCGACGCAG
pos5	*pos5*-F: ctaggaggattatgagatgAGTACGTTGGATTCACATTCCCTAAA<br>*pos5*-R: ATGTGGCATtacctcctagatccTTAATCATTATCAGTCTGTCTCTTGG
lysA	*lysA*-F: ACTGATAATGATTAAggatctaggaggtaATGCCACATTCACTGTTCAGCACCGA<br>*lysA*-R: gcctggagatccttactcgagtttggatccTTAAAGCAATTCCAGCGCCAGTAAT

References

[1] J. Ye, J. Yu, Y. Zhang, M. Chen, X. Liu, S. Zhou, Z. He, Appl Catal B. 2019, 257, 117916.

[2] M. Chen, X. Zhou, Y. Yu, X. Liu, R. Zeng, S. Zhou, Z. He, Environ Int. 2019, 127, 353.

[3] J. Guo, M. Suástegui, K. K. Sakimoto, V. M. Moody, G. Xiao, D. G. Nocera, N. S. Joshi, Science 2018, 362, 813.

[4] J. H. Park, S. H. Lee, G. S. Cha, D. S. Choi, D. H. Nam, J. H. Lee, J. K. Lee, C. H. Yun, K. J. Jeong, C. B. Park, Angew. Chem., Int. Ed. 2015, 127, 983.

[5] Z. Jiang, B. Wang, J. C. Yu, J. Wang, T. An, H. Zhao, H. Li, S. Yuan, P. K. Wong, Nano Energy 2018, 46, 234.

[6] L. Zhang, G. Morello, S. B. Carr, F. A. Armstrong, J. Am. Chem. Soc. 2020, 142, 12699.

[7] Y. Honda, Y. Shinohara, H. Fujii, Catal. Sci. Technol. 2020, 10, 6006.

[8] X. Liu, F. Kang, C. Hu, L. Wang, Z. Xu, D. Zheng, W. Gong, Y. Lu, Y. Ma, J. Wang, Nat. Chem. 2018, 10, 1201.
